# Sex Chromosomes and Sex Phenotype Contribute to Biased DNA Methylation in Mouse Liver

**DOI:** 10.3390/cells9061436

**Published:** 2020-06-09

**Authors:** Qinwei Kim-Wee Zhuang, Jose Hector Galvez, Qian Xiao, Najla AlOgayil, Jeffrey Hyacinthe, Teruko Taketo, Guillaume Bourque, Anna K. Naumova

**Affiliations:** 1 Department of Human Genetics, McGill University, Montréal, QC H3A 1C7, Canada; qw.zhuang@mail.mcgill.ca (Q.K.-W.Z.); najla.alogayil@mail.mcgill.ca (N.A.); 2 Canadian Centre for Computational Genomics, Montréal, QC H3A 0G1, Canada; jose.hector.galvez@computationalgenomics.ca; 3 Department of Biostatistics, Harvard School of Public Health, Boston, MA 02115, USA; qianxiao@hsph.harvard.edu; 4 Department of Quantitative Life Sciences, McGill University, Montréal, QC H3A 0G4, Canada; Jeffrey.Hyacinthe@mail.mcgill.ca; 5 The Research Institute of the McGill University Health Centre, Montréal, QC H4A 3J1, Canada; teruko.taketo@mcgill.ca; 6 Department of Surgery, McGill University, Montréal, QC H4A 3J1, Canada; 7 Department of Obstetrics and Gynecology, McGill University, Montréal, QC H4A 3J1, Canada

**Keywords:** sexual dimorphism, DNA methylation, gene expression, mouse liver, sex-chromosome complement, whole genome bisulfite sequencing

## Abstract

Sex biases in the genome-wide distribution of DNA methylation and gene expression levels are some of the manifestations of sexual dimorphism in mammals. To advance our understanding of the mechanisms that contribute to sex biases in DNA methylation and gene expression, we conducted whole genome bisulfite sequencing (WGBS) as well as RNA-seq on liver samples from mice with different combinations of sex phenotype and sex-chromosome complement. We compared groups of animals with different sex phenotypes, but the same genetic sexes, and vice versa, same sex phenotypes, but different sex-chromosome complements. We also compared sex-biased DNA methylation in mouse and human livers. Our data show that sex phenotype, X-chromosome dosage, and the presence of Y chromosome shape the differences in DNA methylation between males and females. We also demonstrate that sex bias in autosomal methylation is associated with sex bias in gene expression, whereas X-chromosome dosage-dependent methylation differences are not, as expected for a dosage-compensation mechanism. Furthermore, we find partial conservation between the repertoires of mouse and human genes that are associated with sex-biased methylation, an indication that gene function is likely to be an important factor in this phenomenon.

## 1. Introduction

Mammalian males and females carry different sex-chromosome complements (XX in females and XY in males); produce different levels of sex hormones; have different anatomy and physiology; and, in humans, have different risks for developing certain diseases. Gene expression is a major player in defining cell phenotype and is regulated by epigenetic factors, such as DNA methylation and chromatin organization. Hence, it is logical to hypothesize that the difference between the male and female cells results from differences in gene expression, which are accompanied by differences between the male and female cell epigenomes.

In both mice and humans, the expression levels of a subset of genes vary between males and females in somatic cells [1,2,3,4,5]. The gonadal sex of the mouse has a major effect on gene expression levels in mouse liver [1,6]. In contrast, the sex-chromosome complement drives sex-biased gene expression in mouse embryonic stem cells, adult mouse thymus or heart, as well as human peripheral blood cells [2,3,4,7]. Thus, data from both mouse and human studies suggest a complex regulation of sexually dimorphic gene expression where the roles of gonadal sex hormones and sex chromosomes vary between different tissues and developmental stages.

DNA methylation or chromatin modifications are essential regulatory layers involved in the orchestration of gene expression. They also contribute to the sex-biased gene expression and, thereby, sexual dimorphism in phenotypes. Indeed, in humans, global levels of DNA methylation and methylation of repetitive elements, in particular, are higher in males [8,9,10]. In contrast, most differentially methylated regions (DMRs) located within autosomal genes show higher methylation levels in females [8,11,12,13]. Comparison of DNA methylation patterns in individuals with numerical sex-chromosome aberrations (45,X females with Turner syndrome and 47,XXY males with Klinefelter syndrome) suggest a strong effect of sex-chromosome dosage on the human methylome [14,15,16,17]. In mice, DNA methylation levels tend to be higher in females, with about twice as many hypermethylated DMRs found in females than in males [18]. Furthermore, combined data from the ENCODE and Roadmap Epigenomics projects suggest that sex differences in epigenetic marks are more likely to occur at enhancer regions and “bivalent” regions that carry both active and repressive epigenetic marks [6,19]. Thus, DNA methylation and chromatin modification data from the two best studied mammalian species support the notion of a sexually dimorphic epigenome. However, the exact genetic and molecular mechanisms responsible for these sex biases and the distinct contributions of sex-chromosome linked genes versus gonadal sex hormones have only recently begun to be elucidated.

To better understand how sex-biased DNA methylation patterns arise and whether they are associated with sex-biased expression, we undertook a survey of DNA methylation and gene expression in the liver of adult mice with different combinations of sex-chromosome complement and phenotypic sex: XY males (XY.M), XX females (XX.F), females with monosomy X (XO.F), and sex reversed XY females (XY.F) (Figure S1, Table S1). We generated catalogues of those DMRs that depended on the sex phenotype and those that depended on the sex-chromosome complement. For simplicity, both types of DMR are referred to as sex-associated DMRs (sDMRs) from this point on. We present evidence that the sex phenotype and sex-chromosome complement are both major contributors to sexual dimorphism in DNA methylation. We also compared our mouse data with a human liver methylation dataset and found overlaps in the sex-biased methylation patterns.

## 2. Materials and Methods

### 2.1. Mouse Strains and Crosses

B6.C3H/HeSn-*Paf* mice (referred to as *Paf* from this point on) were generated by backcrossing C3H/HeSn-Paf/J carriers of the patchy fur (*Paf*) mutation purchased from the Jackson Laboratory to C57BL/6J mice (Jackson Laboratory, Bar Harbor, Maine, USA). Males that carry the *Paf* mutation were identified based on their hair loss phenotype and crossed to C57BL/6J females. Female offspring from these crosses were genotyped using reverse transcription followed by PCR (RT-PCR) for the *Xist* gene, which is expressed in XX females, but not in XO females [20,21]. Liver samples from 8-week-old N6 and N7 XO females and their XX*^Paf^* littermates were collected and used for DNA methylation and expression analyses (Figure S1).

The B6.Y^TIR^ mouse (referred to as TIR from this point on) was established by placing the Y chromosome from a variant of *Mus musculus domesticus* caught in Tirano, Italy (TIR) onto the C57BL/6J genetic background [22,23]. TIR males (N69-72) were crossed to C57BL/6J females to produce XY and XX females and XY males. Females were genotyped using PCR for the Y-linked *Zfy* gene [23,24]. For whole genome bisulfite sequencing (WGBS), liver samples from 8-week-old animals (three mice in each of the sex/genotype groups and two mice for the XX*^Paf^*.F group) were used. For validation experiments, between four and eight liver samples from each sex/genotype group were collected. For expression analysis, 4 XX.F, 4 XY.F, 4 XY.M, 5 XX*^Paf^*.F, and 4 XO.F liver samples from 8-week-old mice were collected (Table S1).

All procedures were conducted in accordance with the guidelines set by the Canadian Council on Animal Care (Ottawa, Ontario, Canada) and were approved by the Animal Care Committee of the McGill University Health Center (Montreal, Quebec, Canada).

### 2.2. DNA Extraction and Sequencing

DNA from mouse livers was extracted using a standard proteinase K phenol/chloroform procedure or by QIAamp Fast DNA Tissue Kit (Qiagen, Venlo, Netherlands). Library preparation and WGBS were performed at the McGill University and Genome Quebec Innovation Centre. Paired-end sequencing using an Illumina HiSeqX sequencer was distributed across 17 lanes in three sequencing runs. Each lane had a mix of sequencing libraries, to avoid batch effects. Once demultiplexed, each sample had a minimum of 400 million raw paired reads of 150 bp each.

### 2.3. Methylation Calling and Single Nucleotide Polymorphism (SNP) Filtering

Methylation calling was performed with the GenPipes (v3.1.4) “*Methyl Seq”* pipeline [25]. Briefly, raw reads were trimmed for quality (quality score ≥30) using trimmomatic (v 0.36) [26], and then aligned to the *Mus musculus* GRCm38 (mm10) reference genome using the bisulfate read aligner Bismark (v 0.18.1) [27]. All samples had a mean coverage of at least 20×, across the genome. Duplicate reads were then removed using Picard (v 2.9.0) [28] and methylation calls were extracted using the methylation caller included as part of Bismark. Finally, called CpGs were filtered to remove any sites that overlapped with loci from the NCBI reference SNP database for GRCm38 (dbSNP version 142) [29], to reduce potential bias introduced by genetic variation between samples. On average, this meant the removal of 4.4 million CpGs from each sample.

### 2.4. Detection of Differentially Methylated CpG Sites (DMC) or Sex-Associated Differentially Methylated CpG Sites (sDMC)

Differential methylation was detected using DSS (v2.32.0) [30] and methylKit (v1.10.0) [31]. The quality control (QC) step was done by checking the distributions of CpG methylation levels and the CpG coverages. The methylation calls were preprocessed by removing CpG sites with coverage >500× to account for PCR bias. A principal component analysis (PCA) was performed on the methylation levels of the top 2500 most variable CpG sites. A heatmap on the top 2500 most variable CpG sites was generated with ComplexHeatmap (v3.10) [32], using Spearman correlation to cluster loci based on methylation levels. Methylation level was in the range of 0% to 100%, where 0% meant no methylation and 100% represented full methylation.

DSS applied a 500-bp (default) smoothing window to estimate and compare methylation of CpG sites across conditions. The smoothing process was done by the function *DMLtest*, implemented in the DSS package. After the smoothing step, Wald tests were used for testing differential methylation at each CpG site. The function *callDML* then took the result from *DMLtest* as input to extract differentially methylated CpG sites (DMC) or sex-associated differentially methylated CpG sites (sDMC) by applying a default threshold *p*-value < 1 × 10^−5^. Subsets of DMC or sDMC were then tailored based on methylation differences. The intersections among DMC or sDMC in different comparisons were illustrated by UpSet Plots using the R package UpSetR (v1.4.0) [33,34].

### 2.5. Detection of Sex-Associated Differentially Methylated Regions (sDMRs)

Sex-associated differentially methylated regions (sDMRs) were estimated to leverage the spatial correlation of methylation levels among consecutive CpG sites. With the DSS tool, similar to *callDML*, the core function *callDMR* took the result from *DMLtest* as input. Multiple restrictions were applied, including a requirement that each sDMR had a minimum length of 50 bp and spanned at least three CpG sites. Any two sDMRs within 100 bp from each other were merged into one bigger sDMR. Only sDMRs with methylation differences >20% were kept as the output of DSS.

We also identified sDMR using methylKit, where the boundaries were identified by tiling the genome with 300 bp length and 300 bp step-size windows. Only CpGs with sequencing depth in the range [10X, 500X] were considered and only tiles with at least one CpG site were used. Differentially methylated tiles were detected with *q*-value <0.05, where q-values were adjusted *p*-values using the Success Likelihood Index Method (SLIM) method [35], and with a methylation difference >20%. Over-dispersion correction (correction method “MN” in methylKit) was applied to further limit false positives. If not specified, default values were used for all other parameters implemented in DSS and methylKit. The final sDMR list used in downstream analyses was a union from the results of DSS and methylKit. Only the sDMRs from DSS were kept in the case of overlap.

### 2.6. Pyrosequencing Assays

One thousand nanograms of DNA per sample was treated with sodium bisulfite using EpiTect Bisulfite Kit (Qiagen, NL, USA). Primers for sex-associated differentially methylated regions (sDMRs) (50–70 bp) were designed using the PyroMark Assay Design 2.0 Software (Qiagen, NL, USA). The list of primers is provided in Table S2. Pyrosequencing was carried out using the PyroMark Q24 Advanced platform and PyroMark Q24 Advanced CpG Reagents (Qiagen, NL, USA). The results were analyzed using the PyroMark Q24 Advanced software (Qiagen, NL, USA).

### 2.7. Basic Annotation of sDMR/CpG Sites

Basic annotations, including CpG island (CGI) annotation, genic annotation, and chromatin state estimation, of sDMR or CpG sites were performed for sDMRs from the four comparisons and one background group containing all CpG sites (sequencing depth in the range 10×, 500×) with the R package annotatr (v1.12.1) [36]. The CpG island (CGI) annotation was done using a reference retrieved from the R package AnnotationHub (v2.18.0) [37], where the CpG shores were defined as 2 Kb upstream/downstream from the end of CpG islands and CpG shelves were defined as another 2 Kb upstream/downstream from the farthest limit of CpG shores, while the remaining genomic regions were noted as inter CGI. The genic annotation was performed using R package GenomicFeatures (v1.38.2) [38] and data from TxDb.Mmusculus.UCSC.mm10.knownGene (v3.10.0) [39]. Specifically, the genic annotations included 1–5 Kb upstream of the transcription start site (TSS) (1–5 kb), the promoter (<1 Kb upstream of the TSS), 5′UTR, exons, introns, and 3′UTR. The chromatin state was annotated with a chromatin state estimation dataset [40,41,42] (https://www.encodeproject.org/files/ENCFF580WIS) that had predicted 15 chromatin states of liver genome of male mice (strain C57BL/6J) with chromHMM [43]. The 15 chromatin states included the following: enhancer (Enh), weak enhancer (EnhLo1 and EnhLo2), poised enhancer (EnhPois1 and EnhPois2), heterochromatin (HetCons), heterochromatin repressed by PolyComb (HetFac), quiescent regions (Quies, QuiesG), active TSS (TssA), flanking TSS (TssAFlnk1 and TssFlnk2), bivalent TSS (TssBiv), and strong trancription (Tx1 and Tx2).

### 2.8. Enrichment Analysis of Repetitive Elements

sDMRs were annotated with UCSC track RepeatMasker [44,45,46] for the analysis of repetitive elements (or repeats). We calculated the number of times each repeat family/subfamily overlapped our list of sDMRs. As done before [47], in order to have a random baseline to compare against, for each comparison, we simulated a library of 300 bp random regions, with the same distribution of distance to nearest genes as the sDMRs. For each comparison, the simulation was repeated 1000 times and we counted the incidence of observed count being higher than the random baseline for each repeat family/subfamily. A repeat family/subfamily was identified as over-represented (enriched) when the over-represented incidence >995/1000 (*p* < 5 × 10^−3^).

### 2.9. Motif Enrichment Analysis with Homer

The genomic coordinates of all sDMRs were then used to detect enrichments of previously known transcription factor binding sites with Homer (v.4.9.1) [48], applying the option “-len 8,10” in defining the target motif lengths (targeting motifs with length 8 bp and 10 bp). The top five enriched motifs in each of the four comparisons were presented in the form of heatmaps with the color intensity denoting the enrichment level (in %) of motifs.

### 2.10. RNA Extraction and Sequencing

Total RNA was isolated using TRIzol Reagent (Thermo Fisher Scientific, MA, US) according to the manufacturer’s instructions and followed by purification using the RNeasy MinElute Cleanup Kit (Qiagen, NL). Library preparation and RNA-sequencing (RNA-seq) were performed at the McGill University and Genome Quebec Innovation Centre. Paired-end sequencing using an Illumina NovaSeq 6000S4 sequencer was distributed across two lanes in two sequencing runs. Each lane had a mix of sequencing libraries to avoid batch effects. Once demultiplexed, each sample had a minimum of 100 million raw paired reads of 100 bp each.

### 2.11. Differential Expression Analysis

Differential gene expression analysis was performed using the GenPipes “*RNA-seq*” pipeline [25]. Briefly, reads were trimmed and filtered for quality, then they were aligned to the mouse reference genome (GRCm38) using STAR [49]. The abundance of each transcript was estimated using HT-Seq Count (v.0.6.0) [50]. PC analysis and hierarchical clustering were performed on the abundance data, after doing variance stabilizing transformation with DESeq. Differential gene expression was determined with both the DESeq (v. 1.32.0) [51] and EdgeR (v. 3.22.5) [52] packages.

Additionally, differential transcript expression was estimated using the Kallisto–Sleuth pipeline [53,54]. Briefly, Kallisto (v. 0.44.0) was used to perform pseudo-alignment of trimmed and filtered reads to the mouse reference genome (GRCm38). The results of the pseudo-alignment were then imported to Sleuth (v. 0.30.0) to estimate transcript abundance and perform pairwise comparisons between experimental groups. The TSS of differentially expressed transcripts (adjusted *p*-value below 0.05) was used to calculate the distance to the closest sDMR, using BEDtools (v. 2.26.0) [55].

### 2.12. Analysis of sDMC- or sDMR-Proximal Genes

The proximal genes of genomic loci/regions (sDMC or sDMR) were identified by annotatr (v1.12.1) [36], where the genic annotations included 1–5 Kb upstream of the TSS (1–5 kb), the promoter (<1 Kb upstream of the TSS), 5′UTR, exons, introns, 3′UTR, and intergenic regions. Genomic regions annotated as non-intergenic got assigned with related genes, which were named as sDMC-proximal or sDMR-proximal genes for inputs as genomic loci or regions, respectively.

Associated genes for intergenic sDMR were not included because the confidence level of identifying true sDMR-associated genes was low and inconsistent with that used for identifying sDMC-proximal or sDMR-proximal genes for sDMC or sDMR overlapping with genic regions.

### 2.13. Enrichment of sDMR Near Differentially Expressed Genes (DEG)

sDMR-proximal genes were identified for each sDMR using a distance cutoff of up to 5 Kb upstream of TSS (genic region + 5 kb upstream of TSS). For each comparison (XX.F vs. XY.M and XY.F vs. XY.M), we evaluated the enrichment of DEG in the sDMR-proximal genes using a hypergeometric test with *phyper* function in the R package stats (v.3.6.1) [56,57].

For the overlaps between sDMR-proximal genes and DEG, we also tested the association between the methylation level of sDMR (higher/lower methylations in males) and expression levels of DEG (lower/higher expressions in males) for both XX.F versus XY.M and XY.F versus XY.M comparisons. Chi-square test was applied in testing the significance of higher/lower expression levels of DEG being associated with enrichments of sDMRs with lower/higher methylation.

### 2.14. Orthologous Gene of Human and Mouse

The list of 15,779 orthologous genes (15,212 autosomal genes and 567 genes on the X chromosome) of human and mouse was retrieved from the vertebrate homology section on the Mouse Genome Informatics (MGI) (The Jackson Laboratory, Bar Harbor, MA, USA) (http://www.informatics.jax.org).

### 2.15. Human Datasets

The human high-density lipoprotein (HDL) dataset [58] was generated for studying the impact of sex on DNA methylation. DNA methylation was measured with the Infinium HumanMethylation450 BeadChip. Liver biopsies were collected from an adult (average age of 49 years) obese cohort, consisting of 34 males and 61 females. CpG sites with significant methylation differences (>5%) between sexes (*q* < 0.05), 2582 on the X chromosome and 4192 on autosomes, were used for our analysis of sDMC-proximal genes.

The Turner dataset [14] focused on DNA methylation differences between females with a normal 46,XX karyotype and females with Turner (45,X) syndrome. Peripheral blood mononuclear cells (PBMC) from three 45,XO individuals (aged 11, 13, and 15 years) and three 46,XX individuals (aged 18, 18, and 19 years) were used for DNA methylation analysis. DNA methylation was measured with the Infinium humanmethylation27 beadchip. CpG sites with significant methylation differences (methylation differences >10% and a false discovery rate <5%) were identified. A total of 1194 genes were associated with the 592 DMC between Turner and control females, although the list of differentially methylated CpG sites was not provided in the original paper. We used this list of 1194 sDMC-proximal genes for our comparison of methylation in human and mouse.

### 2.16. Comparison of Sex-Biased Methylation in Human and Mouse Liver

We identified sDMR-proximal genes for sDMR identified in the XX.F versus XY.M comparison of our mouse dataset and sDMC-proximal genes for sDMC from the human HDL dataset. For the Turner dataset, we extracted genes associated with sDMC. We focused on the orthologous genes between humans and mice for all three datasets. We inspected the overlaps of genes between human and mouse datasets. The significance of overlaps was tested with the hypergeometric test with *phyper* function in the R package stats (v.3.6.1) [56,57].

## 3. Results

### 3.1. Sex Phenotype, Sex-Chromosome Complement, and Genetic Background Influence Global DNA Methylation Profiles in Mouse Liver

To dissect the impacts of sex phenotype and sex-chromosome complement on DNA methylation, we used crosses from two strains of mice that show high rates of sex reversal (TIR) or X chromosome monosomy (*Paf*) (Methods). TIR mice carry a sex determining region of Chr Y (*Sry*) gene variant, which is, on the C57BL/6J genetic background, inefficient to upregulate its target Sry-box transcription factor 9 (*Sox9*) gene that is necessary for initiating testicular differentiation [59,60]. This results in about 50% of XY^TIR^ mice developing bilateral ovaries with female phenotype, and the rest developing bilateral testes with male phenotype or unilateral ovary and contralateral testis with intersex external genitalia (true hermaphrodites) [23,61]. The latter group was excluded from our experiments (Figure S1). *Paf* mice carry a *Paf* mutation on the X chromosome. The mutation is located within the boundary of the pseudoautosomal region and interferes with segregation of the X and Y chromosomes [62,63,64]. Therefore, about 20% of the female progeny of the X*^Paf^*Y males are XO females (Figure S1). In our study, N5 and N6 X*^Paf^*Y males were used for the generation of XO females as further backcross onto the C57BL/6J strain lowered the frequency of XO female production among some X*^Paf^*Y progeny (TT, unpublished data).

Liver samples were obtained from XX females (XX.F), XY males (XY.M), and sex-reversed XY females (XY.F) from the TIR cross and XO females (XO.F) and XX*^Paf^* females (XX*^Paf^*.F) from the *Paf* cross (Figure S1, Table S1, Methods). We measured DNA methylation in a total of 14 samples using WGBS. Mice from the *Paf* cross still harbored portions of the genome derived from the C3H strain on a C57BL/6J genetic background and their genetic heterogeneity led to variance in DNA methylation levels. We observed methylation differences between the two groups of XX females, where XX.F had C57BL/6J and XX*^Paf^*.F had mixed genetic background (Figure S2). To minimize the impact of genetic variation, we filtered out CGs overlapping with SNPs (Methods). After CpG methylation calling and SNP filtering, the quality of the WGBS data was assessed for all samples (Methods). Methylation levels were comparable across samples, with around 20% of CpG sites having methylation levels <50% (Figure S3a). Extremely high coverage CpG sites (coverage >500×) were excluded to avoid likely artifacts (Figure S3b).

**Figure 1:**
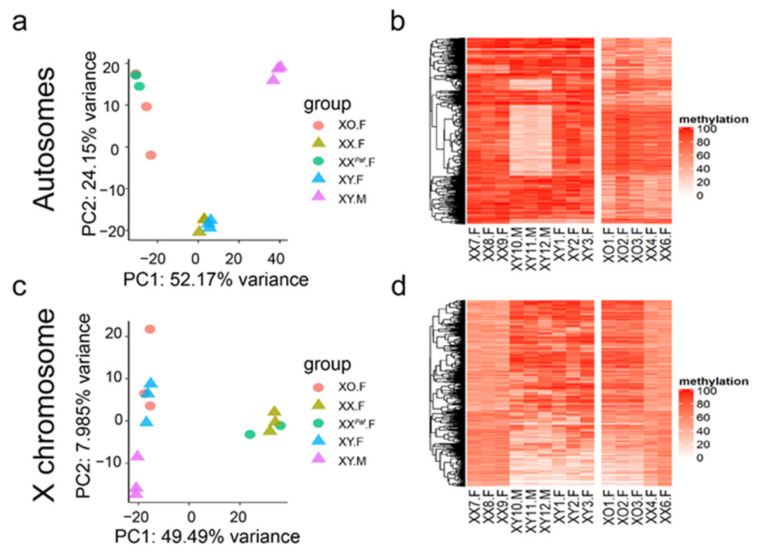
Sex phenotype, sex-chromosome complement, and genetic background influence methylation levels. (**a**,**b**) Principal component analysis (PCA) plot and heatmap show differential methylation across 2500 autosomal CpG sites with the largest variance in methylation. (**a**) Samples form clusters by phenotypic sex and genetic backgrounds of strain crosses. (**b**) Heatmap shows methylation levels of CpG sites for samples from theTIR and *Paf* crosses separately. The generated clustering dendrogram is then used to guide plotting the heatmap for the *Paf* cross. (**c**,**d**) PCA plot and heatmap show differential methylation across 2500 X-linked CpG sites with the largest variance in methylation. (**c**) Samples form clusters by number of X chromosomes and phenotypic sex. (**d**) Heatmap shows methylation levels of CpG sites for samples from the TIR and *Paf* crosses separately. Higher color intensity represents higher methylation level.

Next, we performed PCA of the methylation levels of the top 2500 most variable CpGs across autosomes and X chromosome separately (Methods). When plotting the top two principal components (PCs) for autosomes, explaining 76.3% of the variance, samples clustered into three main groups: (i) females from the *Paf* cross (XO.F and XX*^Paf^*.F)*,* (ii) females from the TIR cross (XX.F and XY.F), and (iii) males (XY.M) (Figure 1a). The females from the *Paf* cross clustered separately from the females from the TIR cross, which is consistent with what was seen in Figure S2. They also showed higher inter-individual variation. The effects of sex phenotype and genetic background were also visible in a heatmap of the same 2500 CpG loci (Figure 1b). Males (XY.M) differed from the females from both crosses with clusters of CpG sites with lower methylation levels. Females from the *Paf* cross showed relatively lower methylation levels than the females from the TIR cross. When plotting the top two PCs based on CpGs on the X-chromosome, explaining 57% of the variance, samples clustered into two main groups: females with two X chromosomes (XX.F and XX*^Paf^*.F) and mice with one X (XY.F, XY.M and XO.F). It is worth noting that males (XY.M) tend to cluster separately from females with one X (Figure 1c). The effect of genetic background was not as pronounced in the methylation of CpGs on the X chromosome (Figure 1c,d).

### 3.2. Identification of Sex-Associated Differentially Methylated CpGs (sDMC)

**Figure 2:**
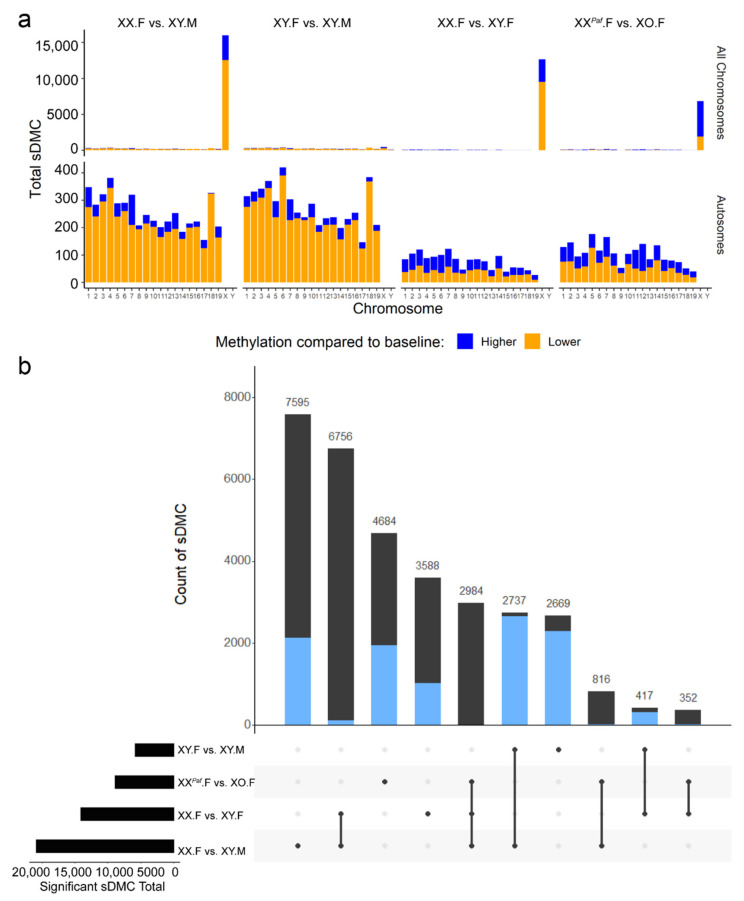
Phenotypic sex and sex-chromosome complement contribute to sex bias in DNA methylation. (**a**) Counts of sex-associated differentially methylated CpG sites (sDMC) per chromosome in each comparison. Top row shows the distribution of sDMC across all chromosomes. Bottom row shows the autosomal sDMC. The *x*-axis represents chromosome ID, and the *y*-axis represents the number of sDMC. Orange portion of the bar corresponds to sDMC with lower methylation compared with baseline. Blue corresponds to sDMC with higher methylation level compared with baseline. Baseline samples are XX.F (XX.F vs. XY.M), XY.F (XY.F vs. XY.M), XX.F (XX.F vs. XY.F), and XX*^Paf^*.F (XX*^.Paf.^*F vs. XO.F). (**b**) UpSet Plot shows the overlap of sDMC detected across comparisons. Each horizontal bar on the left represents the total number of sDMC identified in each comparison. Each vertical bar shows the size of a subset, while the blue and black portions represent sDMC on autosomes and sex-chromosomes, respectively. Different vertical bars are mutually exclusive, and bars with intersection size <50 are not shown.

To identify the CpGs with methylation levels that depended on sex phenotype and/or sex-chromosome complement (referred to as sex-associated differentially methylated CpG (sDMC)), we compared CpG methylation levels in four comparisons: XX.F versus XY.M, XX.F versus XY.F, XY.F versus XY.M, and XX*^Paf^*.F versus XO.F. To minimize the impact of the genetic background on our ability to detect sex-biased DNA methylation, we used the three experimental groups from the TIR cross as the main discovery set and conducted comparisons between XX*^Paf^*.F and XO.F separately. For each comparison, we detected sDMC using the DSS tool (difference >20%, *p*-value < 1 × 10^−5^, Methods) [30]. The comparison between groups with different sex phenotypes, but the same sex-chromosome complement, XY.F versus XY.M, identified more sDMC on autosomes than on the X chromosome (5266 sDMC on autosomes, 465 sDMC on the X chromosome, and 125 sDMC on the Y chromosome) (Figure 2a). In contrast, comparisons between groups with the same sex phenotype, but different sex-chromosome complements, XX.F versus XY.F and XX*^Paf^*.F versus XO.F, identified fewer sDMC on autosomes, but more on the X chromosome (1452 and 2007 on autosomes, 12,650 and 6856 on the X chromosome, and 6 and 4 on the Y chromosome, respectively). The highest number of sDMC was identified in the comparison between groups with both different sex phenotypes and sex-chromosome complements, XX.F versus XY.M (4900 on autosomes, 15,992 on the X chromosome, and 6 on the Y chromosome).

To isolate the effects of sex phenotype and sex-chromosome complement on sex-biased DNA methylation levels, we identified overlaps between sDMC across comparisons for all chromosomes as well as for autosomes only (Figure 2b). The expectation was that sDMC that were common in comparisons with different sex phenotypes (overlap between XX.F vs. XY.M and XY.F vs. XY.M) represented the sex-phenotype dependent sDMC. The overlapping sDMC found in the comparisons with different X-chromosome dosage (XX.F vs. XY.M, XX.F vs. XY.F, and XX*^Paf^*.F vs. XO.F) are controlled by the number of X chromosomes. The comparison XX.F versus XY.M had 7595 unique sDMC (2123 on autosomes, 5469 on the X chromosome, and 3 on the Y chromosome) not found in other comparisons. Further, 2737 of the XX.F versus XY.M sDMC (2644 on autosomes, 92 on the X chromosome, and 1 on the Y chromosome) were also found in the XY.F versus XY.M comparison (different sex phenotypes), whereas 6756 (111 on autosomes, 6643 on the X chromosome, and 2 the Y chromosome) of the sDMC from the XX.F versus XY.M were found in the XX.F versus XY.F comparison (different sex-chromosome complement). A total of 816 sDMC (15 on autosomes and 801 on the X chromosome) overlapped between the XX.F versus XY.M and XX*^Paf^*.F versus XO.F comparisons. A total of 2984 sDMC (2 on autosomes and 2982 on the X chromosome) were shared across the three comparisons with different X-chromosome dosage (XX.F vs. XY.M, XX.F vs. XY.F, and XX*^Paf^*.F vs. XO.F).

X-chromosome dosage is associated with X-inactivation and different methylation levels on the active and inactive X chromosomes. To separate the effect of X-inactivation on X-linked loci from other mechanisms, we analyzed autosomal and X-linked sDMC separately (blue and black bars in Figure 2b). The largest overlap among autosomal sDMC was observed between the two comparisons of groups with different sex phenotypes (XX.F vs. XY.M and XY.F vs. XY.M), while the largest overlaps among X-linked sDMC were found across the three comparisons with different X-chromosome dosage, as expected. In summary, in mouse liver, the impact of the sex phenotype on methylation is more pronounced on autosomes, while the sex-chromosome complement mostly, albeit not exclusively, influences methylation of X-linked loci.

### 3.3. Sex Phenotype and Sex-Chromosome Complement are Responsible for Sex-Associated Differentially Methylated Regions (sDMR)

**Figure 3:**
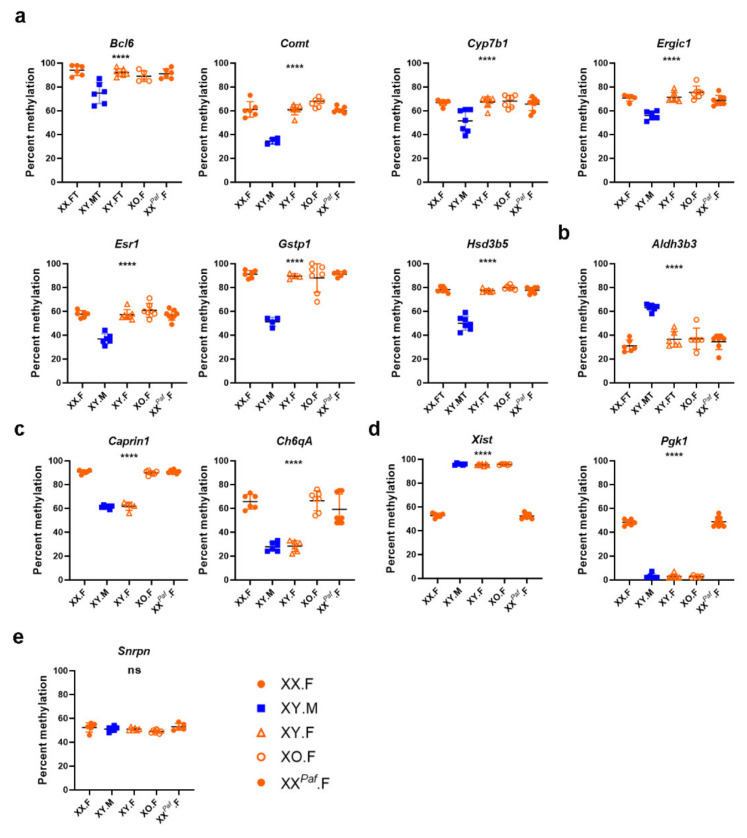
Validation of sex-associated differentially methylated regions (sDMRs) using pyrosequencing assays. (**a**) Autosomal sDMRs with higher methylation in females (*Bcl6*, *Comt*, *Cyp7b1*, *Ergic1, Esr1*, *Gstp1,* and *Hsd3b5*). (**b**) Higher methylation in males (*Aldh3d3*). (**c**) Lower methylation in carriers of an Y chromosome (*Caprin1* and Ch6qA1). (**d**) X-linked sDMRs (*Xist* and *Pgk1*) where methylation levels depend on X-chromosome dosage. (**e**) Methylation levels at the imprinted *Snrpn* DMR are similar across all groups. Each point corresponds to one DNA sample. Error bars show standard deviation, asterisks denote statistically significant differences in methylation levels between groups (one-way analysis of variance (ANOVA)): ****p < 0.0001, ns—not significant.

The specificity of sDMC identification and the functional significance of individual DMC is a subject of debate; however, it is well established that DMRs encompassing multiple CpG sites may be associated with changes in gene expression and chromatin reorganization [65,66]. Hence, we applied DSS [30] and methylKit [31] to characterize sDMRs for the same four pairwise comparisons (Methods). To validate the sDMR detection results, we selected 10 autosomal sDMRs that were identified in at least one comparison and did not reside within CpG islands or repetitive elements (Table S2). Eight sDMRs with methylation levels depending on the sex phenotype were selected: seven with higher methylation in females (*Bcl6, Comt*, *Cyp7b1*, *Ergic1*, *Esr1*, *Gstp1*, and *Hsd3b5*) (Figure 3a) and one with higher methylation in males (*Aldh3b3*) (Figure 3b). We also tested two autosomal sDMRs with methylation levels depending on the sex-chromosome complement (*Caprin1* and Ch6qA1) (Figure 3c) and two X-linked sDMRs that are known to be differentially methylated on the active and inactive X chromosomes, and hence depending on X-chromosome dosage (Figure 3d). Finally, a parental-origin dependent DMR in the promoter of imprinted gene small nuclear ribonucleoprotein N (*Snrpn*) was used as a control, where 50% methylation independent of the sex or sex-chromosome complement was expected (Figure 3e). We performed pyrosequencing methylation assays using additional DNA samples from the same five groups of mice (*n* = 4–8 per group). We observed the expected hypermethylation of the *Xist* and hypomethylation of the *Pgk1* promoters in mice with a single X, and the opposite patterns in mice with two X chromosomes. Interestingly, several autosomal sDMRs identified by one of the packages (*Comt* and *Bcl6*) or only one of the comparisons (*Ergic1* and *Esr1*) were confirmed by pyrosequencing as belonging to the sex-phenotype associated category (Figure 3a). In the cases of *Caprin1* and Ch6qA1 sDMRs, lower methylation levels were associated with the presence of the Y chromosome and not X-chromosome dosage as XO females had higher methylation levels compared with XY females (Figure 3c).

The overall distribution of sDMRs across chromosomes (Figure S4) resembled that of the previously identified sDMC (Figure 2a). Specifically, the comparison XY.F versus XY.M, involving only different sex phenotypes, identified 3847 sDMRs on autosomes, but only 146 sDMRs on the X chromosome. In comparisons between groups with different sex-chromosome complements and same sex phenotype (XX.F vs. XY.F and XX*^Paf^*.F vs. XO.F), fewer sDMR were detected on autosomes in total (803 and 650, respectively) than on the X chromosome (1563 and 1472, respectively). The comparison XX.F versus XY.M, with both different sex phenotypes and sex-chromosome complements, identified multiple sDMRs on both autosomes and X chromosome (2853 and 2483, respectively). Similar to the trends observed in sDMC, males showed lower methylation levels in the majority of autosomal sDMRs in comparisons between groups with different sex phenotypes (2521/2853 in XX.F vs. XY.M and 3414/3847 XY.F vs. XY.M), while nearly half of the autosomal sDMR showed lower methylation in mice with a single X in comparisons between groups with the same sex phenotype, but different sex-chromosome complements (784/1563 in XX.F vs. XY.F and 379/650 in XX*^Paf^.*F vs. XO.F).

### 3.4. Enrichment of Repeat Families and Transcription Binding Motifs in sDMRs Differ between Autosomes and the X Chromosome

To characterize sDMRs, we annotated them with respect to (1) the distributions of CpG islands (CGI), genic regions, and chromatin states; (2) repetitive elements; and (3) DNA motifs (Methods). To measure enrichment in any category, we contrasted our findings to a set of random regions extracted from all CpG sites referred to as the background group. More than 70% of autosomal sDMRs were found to overlap with intergenic (inter-CpG) regions in all four comparisons, as well as in the background group (Figure S5a). The XX*^Paf^*.F versus XO.F comparison showed higher percentages of autosomal sDMR in CpG islands, but lower percentage in CpG shelves compared with all other groups. While on the X chromosome, all four comparisons showed higher percentages of sDMR in CpG islands and CpG shelves compared with the background group, with the XX*^Paf^*.F versus XO.F and XY.F versus XY.M comparisons having the highest and lowest percentage, respectively (Figure S5b). When inspecting genic annotations, sDMRs overlapped with exons and promoters more frequently than the background group for both autosomes and the X chromosome (Figure S5c,d). To estimate the chromatin states associated with sDMR, we used a public annotation reference of 15 chromatin states across the liver genome of male mice [40,41,42]. Autosomal sDMRs showed higher frequencies of overlaps with enhancer regions in all four comparisons and with TSS in comparisons with different X-dosage, XX.F versus XY.F and XX*^Paf^*.F versus XO.F, when compared with the background set (Figure S5e). On the X chromosome, higher enrichment of sDMR overlapping with enhancer regions and TSS was observed in all four comparisons compared with the background set (Figure S5f).

**Figure 4:**
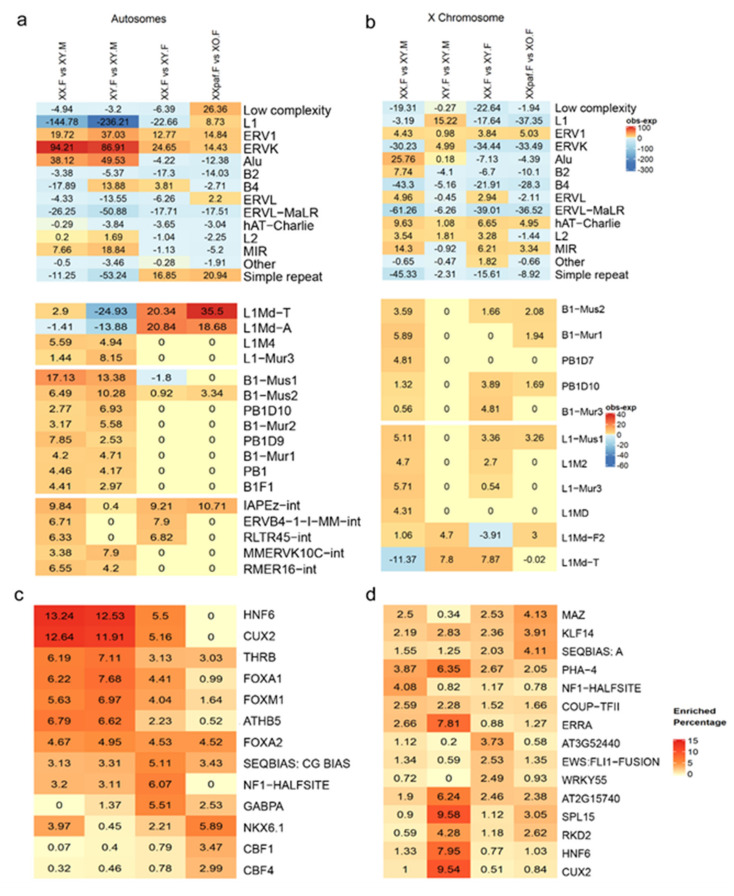
Enrichments of repeat families and transcription factor binding motifs at sDMRs differ between autosomes and the X chromosome. (**a**,**b**) Enrichment of repeats at family and subfamily levels. Each row represents one repeat family/subfamily and the color intensity represents the increased frequency of repeat family/subfamily overlapped with sDMRs (obs) compared with background (exp), the average of 1000 randomly generated sets of 300 bp regions. (**a**) Enrichment of repeats for autosomal sDMR at family (top) and subfamily (bottom) levels. The three selected repeat families from the top to bottom split portions are L1, Alu, and endogenous retrovirus K (ERVK). (**b**) Enrichment of X-linked sDMR with repeats at family (top) and subfamily (bottom) levels, where the two selected repeat families are Alu and L1. (**c**,**d**) Motif enrichment analysis of sDMRs on autosomes (**c**) and on the X chromosome (**d**) using HOMER. The color intensity represents the difference in percentages of sequences with the motif between sDMR and the randomly generated background. The top five motifs from each of the four comparisons are presented and clustered based on the Euclidean distance between the enrichment levels.

Next, we examined the relationship between sDMR and repetitive elements. Repetitive elements are often suppressed via DNA methylation and may contribute to gene regulation [67,68,69]. Therefore, we inspected the distribution of repeats within sDMRs and identified statistically enriched families/subfamilies by comparing them with expected frequencies (Methods). At the repeat family level, long interspersed element-1 (LINE-1/L1) was underrepresented, whereas endogenous retrovirus 1 (ERV1), endogenous retrovirus K (ERVK), and Alu repeats were overrepresented at autosomal sDMR in comparisons between groups with different sex phenotype (XX.F vs. XY.M and XY.F vs. XY.M) (Figure 4a). Two specific subfamilies of repeats (L1Md_T and L1Md_A) in the L1 family were under-represented in the XY.F versus XY.M comparison, but over-represented in comparisons between groups with the same sex phenotype and different X chromosome dosage (XX.F vs. XY.F and XX*^Paf^*.F vs. XO.F) (Figure 4a, bottom panel). The XX.F versus XY.M and XY.F versus XY.M comparisons showed over-representation of multiple subfamilies of Alu repeats among sDMRs, while these repeat families were not over-represented in the XX.F versus XY.F and XX*^Paf^*.F versus XO.F comparisons. One specific subfamily (IAPEz-int) of ERVK showed over-representation in all comparisons between groups with different sex-chromosome complements. In contrast to autosomal sDMRs, sDMRs on the X chromosome showed less repeat enrichment (Figure 4b), even at the level of subfamilies (Figure 4b, bottom panel).

To identify candidate transcription factors potentially associated with sDMR, we conducted motif enrichment analysis with HOMER [48] and plotted the top five most enriched DNA sequence motifs for each comparison (Figure 4c,d). On autosomes, the transcription factor binding motifs for THRB, FOXA2, and CG simple repeats were enriched in all four comparisons (Figure 4c). HNF6, CUX2, FOXA1, FOXM1, ATHB5, and NF1 (half-site) were enriched in comparisons XX.F versus XY.M, XY.F versus XY.M, and XX.F versus XY.F, but not in XX*^Paf^*.F versus XO.F. The XX*^Paf^*.F versus XO.F comparison showed enrichment of GABPA, NKX6.1, CBF1, and CBF4 binding motifs. In contrast, sDMR on the X chromosome showed more uniform motif enrichments, but with lower frequencies, except for motifs for MAZ, KLF14, and SPL15, which were enriched among XX*^Paf^*.F versus XO.F sDMR, and motifs for SPL15, CUX2, ERRA, HNF6, PHA-4, AT2G15740, and RKD2, which were enriched only in XY.F versus XY.M (Figure 4d).

### 3.5. Association between Sex-Associated Methylation and the Transcriptome

**Figure 5:**
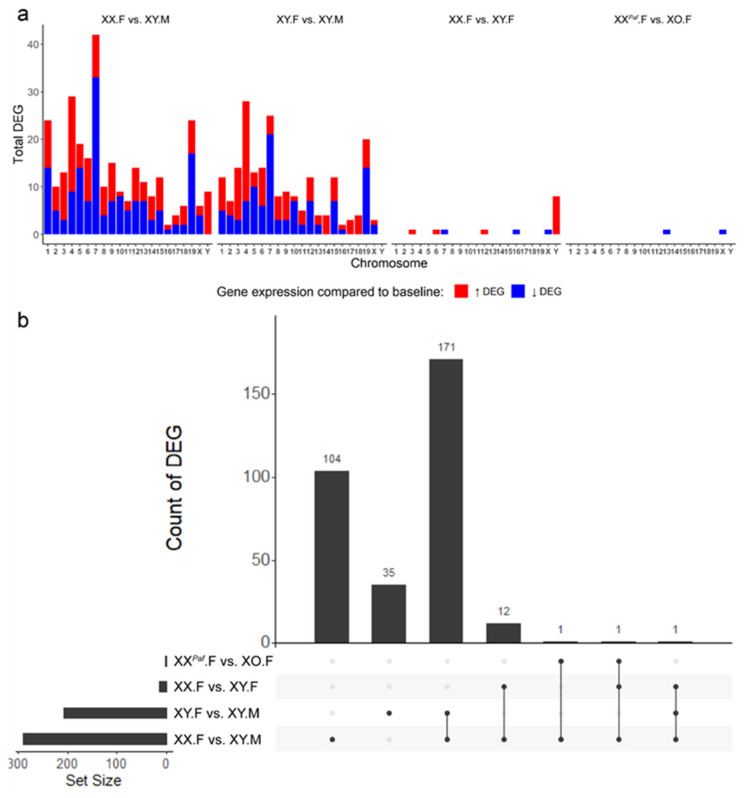
Distribution of differentially expressed genes (DEGs) across chromosomes and overlap of DEG across comparisons. (**a**) Counts of DEG per chromosome in each of the four comparisons. The *x*-axis represents chromosome ID, and the *y*-axis represents the number of DEG on each chromosome. Red portions of the bars correspond to DEG that had higher expression (absolute log_2_ fold change >1.5; adjusted *p*-value <0.05) in the sample compared with baseline, where baseline samples were XX.F (XX.F vs. XY.M), XY.F (XY.F vs. XY.M), XX.F (XX.F vs. XY.F), and XX*^Paf^*.F (XX*^Paf^*.F vs. XO.F). The blue portion represents DEGs that had lower expression in the sample compared with the baseline. (**b**) UpSet Plot shows overlap of DEG detected across comparisons. Each horizontal bar on the left represents the total number of DEGs identified in each comparison. Each vertical bar shows the size of a subset identified in one or multiple comparisons.

To study the association between sex-biased methylation and gene expression, we generated RNA-seq data from the liver of 21 mice from the same five groups (Table S1). After trimming low-quality reads, the quality of the RNA-seq data was assessed. The first two PCs explained a total of 70% of variance and formed four clusters by the groups XO.F, XY.F, XY.M, and XX.F (both from the *Paf* and TIR crosses) with a clear separate cluster of phenotypic males (Figure S6a). A similar result was observed with hierarchical clustering using Euclidean distance between gene expression levels (Figure S6b). To more directly investigate the sex-biased transcriptomes, we performed a differential expression analysis for each pairwise comparison (Methods). Using a cutoff of 1.5 log fold change and 0.05 adjusted *p*-value, we identified 290 differentially expressed genes (DEGs) in the comparison XX.F versus XY.M, 207 DEGs in XY.F versus XY.M, 14 DEGs in XX.F versus XY.F, and 2 DEGs in XX*^Paf^*.F versus XO.F (Figure 5a, Figures S7 and S8 and Table S3). In general, more DEGs were detected in comparisons between groups with different phenotypic sex (XX.F vs. XY.M and XY.F vs. XY.M) than between groups with the same phenotypic sex, but different sex-chromosome complements (XX*^Paf^*.F vs. XO.F and XX.F vs. XY.F), which is consistent with the relative numbers of sDMR detected. Larger portions of DEGs showed lower expression levels on chromosome 5, 7, 10, 12, 15, 19, and X in males in the XX.F versus XY.M and XY.F versus XY.M comparisons, while DEGs on Y chromosomes all were detected only in Y chromosome carriers in both XX.F versus XY.M and XX.F versus XY.F comparisons (Figure 5a). Out of the 290 DEGs in the XX.F versus XY.M comparison, 172 overlapped with DEGs in the XY.F versus XY.M, 14 with the XX.F versus XY.F, and 2 with the XX*^Paf^*.F versus XO.F (Figure 5b). On average, the ratio of numbers of DEGs per sDMR (DEG/sDMR) per chromosome was between 0.01 and 0.30 on autosomes, while the ratio was near 0 on the X chromosome in the comparisons with different X-chromosome dosage (Figure S9).

For the two comparisons that had a sufficient number of DEGs (XX.F vs. XY.M and XY.F vs. XY.M), we checked the cumulative distribution of distances between their TSS and the nearest sDMR (Methods). We found that sDMRs were particularly enriched around the TSS of transcripts that were significantly over-expressed in phenotypic males (XY.M) (Figure S10). The XX.F vs. XY.M comparison also presented an enrichment of sDMRs around the TSS of transcripts that were both over and under-expressed in phenotypic males. To further investigate the association between sex-biased methylation and expression, we used a distance cut-off of up to 5 Kb upstream of TSS (Methods) and identified 1870 sDMR-proximal genes in the XX.F versus XY.M comparison and 1996 sDMR-proximal genes in the XY.F versus XY.M comparison and compared them to the list of DEGs to evaluate the enrichment of DEGs in sDMR-proximal genes (Methods). For the XX.F versus XY.M comparison, we found that 70 (out of 290) DEG overlapped with sDMR-proximal genes (hypergeometric test, expected number: 10.86, *p* < 2.2 × 10^−16^). For the XY.F versus XY.M comparison, 51 (out of 207) DEGs overlapped with sDMR-proximal genes (hypergeometric test, expected number: 5.29, *p* < 2.2 × 10^−16^) (Table S4). Specifically, in XY.M of the XX.F versus XY.M comparison, 42 genes showed lower methylation and higher expression, 21 genes showed higher methylation and lower expression, 4 genes showed lower methylation and lower expression, and 1 gene showed higher methylation and higher expression (Chi-square test, *p* < 2.511 × 10^−11^). In XY.M of the XY.F versus XY.M comparison, 37 genes showed lower methylation and higher expression, 9 genes showed higher methylation and lower expressions, 2 genes showed lower methylation and lower expression, and no gene showed higher methylation and higher expression (Chi-square test, *p* < 1.478 × 10^−8^). While intergenic and distant regulatory DNA elements have a significant impact on transcriptional regulation, the confidence of identifying proximal genes for intergenic sDMR was low. Therefore, the association between intergenic sDMR and sex-biased transcription was not investigated using the hypergeometric test or the Chi-square test.

### 3.6. Partial Conservation of Sex-Associated Methylation in Proximity of Mouse and Human Orthologous Genes

To determine the functionality of sex-biased DNA methylation found in our crosses, we compared our mouse liver dataset with two publicly available human DNA methylation datasets. The expectation was that functionally important sex bias in DNA methylation would be conserved in humans and associated with orthologous genic regions in a tissue-specific fashion. Therefore, we used genes as an anchor point in our analyses. The human HDL dataset explored sex differences in the liver methylome [58]. The human Turner dataset included a comparison of DNA methylation levels in PBMC in females with monosomy X and females with a 46,XX karyotype [14]. The differences of methylation profiling platforms (WGBS in our mouse dataset and Infinium Human Methylation 450K or 27K BeadChip in the human datasets) were another reason for using a gene-centered approach in the analyses. For that purpose, a repertoire of 15,779 mouse–human orthologous genes was retrieved from the Mouse Genome Informatics (MGI) database. We also identified sDMR-proximal genes (in mouse) and sDMC-proximal genes (in human) (Methods) [70]. In total, we retrieved 2678 human genes from the HDL dataset (2128 on autosomes and 550 on the X chromosome) and 648 genes from the Turner dataset (361 on autosomes and 287 on the X chromosome). Among the mouse datasets, we selected the XX.F versus XY.M comparison (1490 genes, 1087 on autosomes and 403 on the X chromosome), and the XX*^Paf^*.F versus XO.F comparison (706 genes, 313 on autosomes, and 393 on the X chromosome) as biologically the most relevant for comparing to the human HDL and Turner datasets, respectively.

**Figure 6:**
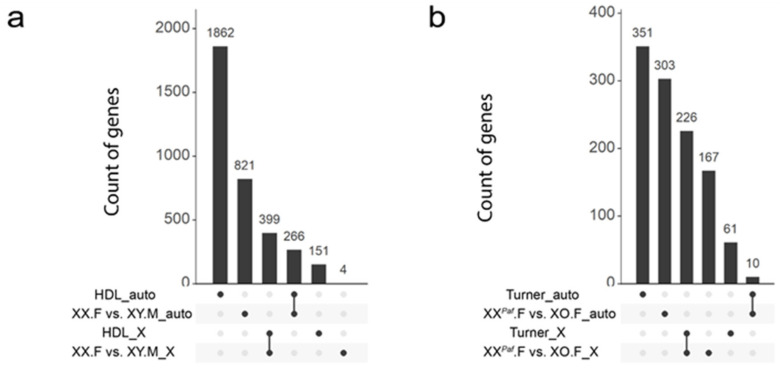
Mouse and human datasets show significant overlap of orthologous genes associated with differential methylated cytosines. UpSet Plot shows the overlap of sDMR-proximal orthologous genes across comparisons. Each horizontal bar on the left represents the total number of sDMR-proximal orthologous genes identified in each comparison. (**a**) Overlaps of sDMR-proximal orthologous genes between the mouse and the human HDL datasets. The top two rows represent genes on autosomes and the bottom two rows represented genes on the X chromosome. (**b**) Overlap of sDMR-proximal orthologous genes between the XX*^Paf^*.F versus XO.F comparison and the human Turner dataset. The top two rows represent genes on autosomes and the bottom two rows represent genes on the X chromosome.

Next, we compared the list of genes from the mouse to those identified in the human datasets. XX.F versus XY.M genes were compared to the HDL dataset (Figure 6a) and XX*^Paf^*.F versus XO.F genes were compared to the Turner dataset (Figure 6b). Notably, among the 706 autosomal sDMR-proximal genes in the XX.F versus XY.M comparison of the mouse dataset, 266 genes overlapped with those found in the HDL dataset (hypergeometric test, expected number 152.06, *p* < 2.2 × 10^−16^, Methods) (Figure 6a). Among the 403 X-linked mouse genes, 399 overlapped with orthologs found in the HDL dataset (hypergeometric test, expected number 390.92, *p* < 5.18 × 10^−5^) (Figure 6a). Among the 313 autosomal sDMR-proximal genes from the XX*^Paf^*.F versus XO.F comparison, only 10 genes overlapped with orthologs identified in the Turner dataset (hypergeometric test, expected number 7.43, *p* < 0.21) (Figure 6b). Among the 393 sDMR-proximal genes on the X chromosome, 226 overlapped with orthologs in the Turner dataset (hypergeometric test, expected number 198.93, *p* < 5.74 × 10^−7^) (Figure 6b). Taken together, the mouse and human liver samples showed significant overlaps of sDMC-proximal genes on both autosomes and X chromosome, while the datasets with different X-chromosome dosage and from different cell types (mouse liver and human PBMC) showed significant overlaps on the X chromosome, but not autosomes.

## 4. Discussion

### 4.1. Methodology for Identifying sDMC and sDMR and Its Impact on Results

In this study, we used two tools to identify regions with different methylation levels: DSS for identifying sDMC and both methylkit and DSS for identifying sDMR. Here, we discuss some caveats that require caution in interpreting the sDMC and sDMR results. To estimate DMC, statistical significances of millions of CpG sites having different methylation levels between groups were assessed independently. In contrast to the large number of statistical tests needed, the sequencing coverage and numbers of samples sequenced for each group is limited by the cost of WGBS. One common practice in response to such a challenge is to leverage the strong spatial correlation of methylation levels among CpG sites nearby. By default, DSS applied a 500 bp smoothing window to allow CpG sites with insufficient sequencing coverage (depth), borrowing information from nearby CpG sites with higher coverages. By nature, the choice of the smoothing window size is one source of selection bias. Estimation of sDMR is a better compromise by reducing the degrees of freedom (i.e., the number of statistical tests required). DSS and methylKit have distinct approaches in defining the boundaries of DMR. DSS uses CpG sites as candidate boundaries and merges nearby sDMC/sDMR, while methylKit uses pre-defined tiles for grouping spatially close CpG. The selection of a sweet spot for merging nearby sDMC/sDMR in DSS and the size of tiles applied in methylKit are hence also sources of potential selection bias for sDMR identification. For instance, the selection of the size of pre-defined tiles can dramatically influence the number of resulting sDMR. The threshold of methylation differences is another parameter that can be tweaked in detecting sDMR. Therefore, we used a 20% threshold for methylation differences, which has been used by other groups in their studies of sex-biased methylation in mice [18,71].

In our study, validation experiments conducted on a small subset of sDMR found no false positive results. However, several results were false negative. Indeed, a number of sDMRs identified in one of the comparisons were validated by pyrosequencing in other comparisons despite the negative result of the WGBS analysis (Figure 3). This has significant implications for the interpretation of results from genome-wide methylation analyses and suggests that, while positive identification of sDMR is robust and may be used as a basis for follow-up studies, negative results are less conclusive and have to be taken with a grain of salt. A generalized scheme for the ensemble of multiple sDMR tools, such as DSS and methylKit, is valuable and warrants further investigation.

### 4.2. Sex Phenotype and Sex-Chromosome Complement Shape Sex-Biased DNA Methylation Patterns

Sexual dimorphism in mammalian phenotypes and gene expression levels are often attributed to the action of gonadal sex hormones [72]. However, in the last decade, several studies demonstrated that sex chromosomes also contribute to sex-biased gene expression or sexual dimorphism in phenotypes [3,72,73,74,75]. To better delineate the distinct influences of the sex phenotype and sex chromosomes on genome-wide patterns of DNA methylation and gene expression in mouse liver, we used a set of mice with different combinations of phenotypic and genetic sexes. We then conducted binary comparisons of methylation levels, as this approach was best suited for our dataset with small numbers of samples per sex/genotype group. Our dataset includes females and males with the same genetic composition, but different gonadal sexes, which allows separating the effects of the sex-chromosome complement (presence of the Y chromosome or X-chromosome dosage) from that of gonadal sex. It also includes females with monosomy X (XO.F), which allows distinguishing the effects of X-chromosome dosage from the impact of the Y chromosome among sex-chromosome complement-dependent sDMRs. Using this experimental design, we demonstrate that differences in DNA methylation levels between XX female and XY male mice are the result of action of at least three factors rather than a single one: the sex phenotype, the X-chromosome dosage, and the presence of the Y chromosome. All three contribute to the sexual dimorphism in the epigenome, with each of them having their own repertoire of target loci.

We find thousands of autosomal and only 146 X-linked sDMR whose methylation depends on the sex phenotype of the mouse. These findings are consistent with other studies of sex-biased DNA methylation in mouse liver implicating testosterone as the strongest influence on autosomal DNA methylation and X-chromosome inactivation as the major influence on the methylation patterns of X-linked loci [6,18,71,76]. Furthermore, our data show that autosomal gene expression patterns mirror the sex-biased DNA methylation patterns in comparisons between phenotypic females and males. However, comparisons of females with different genetic sex (XX.F vs. XY.F) yield very few DEGs (Figure 5a). This rather limited the influence of the sex-chromosome complement on the liver transcriptome is in striking contrast to its prominent effects on intestinal lipid metabolism [75], the transcriptomes of the mouse thymus [2] and heart [7], or the methylation and expression patterns in human blood cells [4,15]. There is also a remarkable difference between autosomal and X-linked sDMR with respect to their relationship with DEGs. For autosomes, we find association between sex bias in expression and methylation; however, the abundance of X-linked sDMR does not translate into sex bias in the expression of X-linked genes (Figure 5b and Figures S7−S9). Our data suggest that the *sex-phenotype dependent sDMRs promote sex differences in gene expression* for autosomal genes represented by equal number of alleles in both sexes, whereas *most X-linked sDMR reflect dosage compensation for X-linked genes that attenuates rather than amplifies sex differences in expression*, an observation consistent with current understanding of the function of X-inactivation [77,78].

The Y-chromosome dependent DMC group consisted of 6643 X-linked, 113 autosomal, and 2 Y-linked sDMC. The caveat here is that the tools used to detect DMC have a number of limitations, as discussed above, and further analyses and validation are necessary to better understand the underlying mechanism. Indeed, the mouse Y chromosome harbors several interesting gene candidates encoding proteins involved in transcriptional regulation or chromatin remodeling that are also expressed in the mouse liver.

Thus, the ensemble of our data and data from other studies shows that, in liver, the sex phenotype is the strongest influence on sex differences in autosomal gene expression and DNA methylation levels with the largest repertoire of target genes on autosomes. Moreover, our data for mouse liver provide little support for the role of epigenetic memory of the embryonic states as a major contributor to the sex bias in autosomal DNA methylation [73]. In contrast, X-chromosome inactivation that takes place early in embryonic development is indeed responsible for the X-dosage dependent DNA methylation of X-linked loci in mouse liver.

### 4.3. Genetic Variation Influences DNA Methylation

Genetic variation has a major impact on DNA methylation in humans [79,80,81,82]. Mouse studies that have assessed the impacts of both genetic variation and sex using different inbred mouse strains show that genetic background also influences methylation levels in mice [18,83]. Our data suggest that, in our mice from the *Paf* cross, portions of the C3H genome that were present on a largely C57BL/6J background led to increase in inter-individual variation in DNA methylation that was most pronounced in large regions of chromosomes 1, 5, and 10. Moreover, filtering of SNPs has not completely alleviated genetic influences on methylation, which suggests, as expected, that there is more to genetic variation than SNPs (Figure 1 and Figure S2). Although less is known about genetic effects on sex-biased methylation in other species of vertebrates, one example from studies on chicken supports genetic influence on sex-biased methylation: two different breeds have their own sex-biased autosomal DNA methylation patterns, but no sex differences that would be common in both breeds [84].

### 4.4. Sex-Biased DNA Methylation of Repetitive Elements

About 60% of all mouse liver sDMR overlap with repetitive elements. We find enrichment of the Alu-repeat family and depletion of L1-Md-T and L1-Md-A families among autosomal sDMRs associated with sex phenotype (Figure 4). This suggests non-random targeting of genomic regions for sex-biased methylation that likely involves gonadal sex steroid signaling. It would be interesting to explore specific DNA motifs associated with these elements to further try to unravel these mechanisms. Moreover, studying the location of these elements in the genome could also help reveal regions that have sex-biased methylation that differs from the primate lineage given that some of the enriched elements are rodent specific. Similarly, the fact that some ERVK subfamilies were found to be enriched on autosomes (Figure 4) is an indication that strain-specific sex-biased methylation could also be present given the rate of retrotransposon insertions in rodents [85].

### 4.5. Insights into the Molecular Mechanisms Underlying Sex Bias in DNA Methylation

DNA methyltransferases (DNMTs) and methylcytosine dioxygenase ten-eleven translocation (TET) proteins are critical for the establishment, maintenance, or erasure of DNA methylation in different cell types and different developmental stages [86,87]. Higher *DNMT3B/Dnmt3b* RNA levels have been found in the livers of human and mouse females compared with males [88,89]. Moreover, lower expression of *Dnmt1* and *Tet2* was reported in male mice [89]. However, we did not detect significant differences in the levels of *Dnmt1*, *Dnmt3a*, *Dnmt3b*, *Tet1*, *Tet2,* or *Tet3* transcripts in our comparisons, suggesting a mechanism of methylation bias that was independent of the levels of DNA methylation machinery.

Transcription factor motif analysis of sex-phenotype dependent autosomal sDMRs found enrichment of binding sites for transcription factors CUX2 and HNF6 that have sex-biased expression in the mouse liver, in agreement with findings reported by other groups [6,71]. However, on the basis of function, most intriguing is the enrichment for pioneer factors of the forkhead box domain family FOXA1/2 and FOXM1 (Figure 4c). Orthologs of forkhead box domain transcription factors are conserved across all metazoan species and play a role in sexual differentiation in vertebrates [90]. FOXA1 is implicated in active DNA demethylation of its target sites in a lineage-specific fashion [91,92]. As a pioneer factor, it opens condensed chromatin and facilitates recruitment of other transcription factors, including receptors for gonadal sex hormones, to enhancers [91,92,93]. Moreover, FOXA1 binding leads to deposition of H3K4me1/2 at enhancer regions [94,95,96]. Hence, we speculate that enrichment of histone H3K4me1 that has been reported in several other studies [6,19,58] and enrichment of sDMR at enhancer regions that we observe in our data (Figure S5e) may be associated with FOXA1 binding. We speculate that FOXA1 is part of the mechanism leading to sex bias in DNA methylation. Experiments directly testing the sequence of events and roles of other factors in the establishment of sex-biased DNA methylation patterns are necessary to confirm or refute this hypothesis.

Motif analysis of X-dosage dependent X-linked sDMR shows little motif enrichment. The one comparison with same genotype, but different sex phenotypes and no X-inactivation, XY.F versus XY.M, shows enrichment for the same factors that are detected in sex-phenotype dependent autosomal sDMR. The caveat here is that only 146 sex-phenotype dependent sDMRs were identified on the X compared with ~1500 X-dosage dependent sDMRs. Therefore, the striking difference between XY.F versus XY.M and the three other comparisons should be taken with caution.

### 4.6. Sex-Biased Methylation in Different Species

Sex bias in gene expression or DNA methylation in somatic cells has been reported in different vertebrate species, including fish, reptiles, birds, and mammals. In mammals, with most data coming from mouse and human studies, sex bias in methylation is usually associated with sex-biased gene expression [6,15], and this study. Interestingly, this is not the case in the chicken brain, which shows genome-wide sex differences in expression levels that do not align with DNA methylation, except for the male hypermethylated region of the Z chromosome [84].

Here, we compared sex-biased DNA methylation in mouse and human livers and found a significant overlap between sDMR-proximal orthologous genes. This suggests that the mechanisms underlying sex-biased DNA methylation are likely associated with gene function and cell type and are at least partially conserved between humans and mice. The enrichment of X-linked sDMR-proximal genes overlapping in mice and humans is an expected outcome as both mammalian species have X-chromosome inactivation. The lack of significant overlap between autosomal genes identified in the Turner dataset and those identified in the mouse XX*^Paf^*.F versus XO.F comparison may be the result of cell-type specificity of sex-biased methylation as different cell types were analyzed in humans and mice.

The relationship between sex-biased methylation, sex chromosome evolution, sex-biased expression, and sexually dimorphic phenotypes becomes more complex in species that are evolutionarily more distant or where sex determination is modulated by environmental conditions, such as certain species of fish and reptiles [97,98,99,100]. Nevertheless, interesting parallels are found between mammals and certain fish species with respect to sex-biased DNA methylation [101]. For example, females of the threespine stickleback, a species with evolutionarily “young” X and Y chromosomes, have higher methylation levels across the genome compared with males and the largest number of DMC on chromosome 19, which is the sex chromosome in the stickleback [101]. Such a distribution of DMC is reminiscent of those reported in humans and mice, including the current report (Figure 2), with higher methylation on autosomes and abundance of sDMC/DMR on the X chromosome in females. These parallels support the idea that DNA methylation has a role in the evolution of sex chromosomes and dosage compensation mechanisms [101,102].

## Supplementary Materials

The following are available online at https://www.mdpi.com/2073-4409/9/6/1436/s1, Figure S1: Mouse crosses used in the study; Figure S2: Methylation heterogeneity associated with SNPs was reduced after SNP removal in the comparison between the two groups of XX females from Paf and TIR crosses; Figure S3: Comparable distributions of methylation levels and removal of CpG sites with extremely high coverage across all 14 samples; Figure S4: Differential distributions of sDMR related to sex phenotype and sex-chromosome complement; Figure S5: Distributions of sDMR differ between autosomes and the X chromosome in CpG island annotation, genic annotation, and estimation of chromatin states; Figure S6: Quality assessment of RNA-seq data for samples illustrates sex-biased gene expression associated with phenotypic sex and sex-chromosome complement; Figure S7: Differential gene expression analysis with EdgeR shows sex-associated differentially expressed genes, especially in the comparisons XX.F vs. XY.M and XY.F vs. XY.M; Figure S8: Differential gene expression analysis with DESeq confirmed the result from EdgeR that more differentially expressed genes are found in the comparisons XX.F vs. XY.M and XY.F vs. XY.M; Figure S9: Ratio of numbers of DEG over numbers of sDMRs across chromosomes; Figure S10: sDMRs are enriched around the TSS of transcripts that are significantly over-expressed in phenotypic males (XY.M); Table S1: List of samples used in WGBS and RNA-seq experiments; Table S2: List of pyrosequencing assays used for validation; Table S3: List of differentially expressed genes (DEGs); Table S4: DEG, methylation levels of sDMR and sDMR-proximal genes.
